# Case Report: Dynamic Interdependencies Between Complementary and Alternative Medicine (CAM) Practice, Urinary Interleukin-6 Levels, and Fatigue in a Breast Cancer Survivor

**DOI:** 10.3389/fpsyt.2021.592379

**Published:** 2021-06-02

**Authors:** Magdalena Singer, Michaela Ott, Harald R. Bliem, Birgit Hladschik-Kermer, Francisco M. Ocaña-Peinado, Emil Chamson, Christian Schubert

**Affiliations:** ^1^Department of Medical Psychology, Center for Public Health, Medical University of Vienna, Vienna, Austria; ^2^Department of Psychology, University of Innsbruck, Innsbruck, Austria; ^3^Department of Statistics and Operations Research, University of Granada, Granada, Spain; ^4^Department of Translation Studies, University of Innsbruck, Innsbruck, Austria; ^5^Clinical Department of Medical Psychology, Medical University of Innsbruck, Innsbruck, Austria

**Keywords:** breast cancer, cancer survivor, CAM, IL-6, fatigue, time series analysis, qualitative method, integrative single-case study

## Abstract

**Background:** This study investigated the influence of complementary and alternative medicine (CAM) techniques (i.e., Jin Shin Jyutsu, music, physiotherapy, Tai Chi, and energy healing) on urinary interleukin-6 (IL-6) levels and fatigue in a 49-year-old breast cancer survivor suffering from cancer-related fatigue and depression. Data were sampled under conditions of “life as it is lived.”

**Methods:** For 28 days, a female breast cancer survivor collected her full urine output in 12-h intervals from about 8 a.m. to 8 p.m. and from about 8 p.m. to 8 a.m. These urine samples were used to determine urinary IL-6 levels through ELISA and creatinine concentrations via HPLC. In 12-h intervals (every morning and evening), the patient completed the DIARI, which included fatigue measurement and notes on incidents and activities such as CAM practice. In addition, the patient was interviewed weekly to identify meaningful everyday incidents. In this context, CAM practice was also discussed. Time series analysis consisted of ARIMA modeling and cross-correlational analyses (*p* < 0.05).

**Results:** When each CAM technique was considered separately in time series analysis, CAM was consistently associated with increases in urinary IL-6 release and decreases in fatigue. Furthermore, when all CAM techniques experienced as positive were included in one time series, a biphasic urinary IL-6 response pattern was found in which CAM practice was first preceded by decreases in IL-6 by 12–0 h and then followed by increases in IL-6 after 108–120 h. Finally, cross-correlations between IL-6 and fatigue showed that increases in IL-6 were followed by decreases in fatigue intensity after 48–60 h and, conversely, that decreases in fatigue intensity were followed by decreases in IL-6 after 24–36 h and 48–60 h.

**Conclusion:** IL-6 increases and fatigue decreases highlight potential health-promoting effects of CAM practice. Moreover, a cyclic IL-6 pattern in response to all CAM activities experienced as positive underscores that CAM was meaningful to the patient. Additionally, a negative feedback circuit between IL-6 and fatigue intensity was detected. Taken together, this study confirms the necessity of integrating subjective meaning and dynamic complexity into biopsychosocial research in order to understand human functioning under real-life conditions.

## Introduction

Complementary and alternative medicine (CAM) techniques such as Tai Chi, yoga, meditation, imagery, acupuncture, massage, Qigong, and prayer have increasingly attracted attention among cancer patients and survivors (1–4). Eighty to nighty percent of cancer survivors report the use of at least one technique from this broad and diverse range to deal with long-term behavioral symptoms such as fatigue, depression, sleep disturbance, and pain (5, 6). Research has begun to examine the impact of these techniques on pro- and anti-inflammatory biomarkers and, more specifically, on interleukin-6 (IL-6) (7).

IL-6 is a keystone cytokine that has been connected to long-term behavioral symptoms of breast cancer such as fatigue, depression and cognitive disturbances (8, 9). High levels of circulating IL-6 have also been associated with tumor progression/recurrence and shorter survival time for breast cancer patients (10). IL-6 plays a crucial role in pathophysiological conditions for a spectrum of diseases. Furthermore, it regulates homeostatic processes in the central nervous system, the immune system and the metabolism under normal physiological conditions (11–13). IL-6 is a pleiotropic cytokine displaying pro- and anti-inflammatory properties (14–16).

So far, results from studies examining IL-6 response to CAM practice in breast cancer survivors have been heterogeneous. For example, Bower and Irwin (7) conducted a descriptive review of 26 RCT studies investigating the impact of mind-body-therapies (MBTs) (Tai Chi, Qigong, Yoga, and meditation) on inflammation in various populations (breast cancer and other cancer patients, heart failure patients, and healthy subjects). Their review showed inconsistent findings with regard to the effects of MBTs on circulating inflammatory markers, including CRP and IL-6, and on stimulated cellular cytokine production. Interestingly, the studies with non-significant results for inflammatory markers did show beneficial effects on symptoms and other outcomes, indicating a positive therapeutic health effect. More consistent findings were seen in this review for genomic markers, which generally fluctuate less than circulating ones. The seven relevant studies showed decreased expression of inflammation-related genes and reduced signaling through nuclear factor ‘kappa-light-chain-enhancer’ of activated B-cells (NF-κB) (7). NF-κB is a proinflammatory transcription factor strongly linked with IL-6 production (17).

A systematic review by Sanada et al. (18) of 13 articles based on 11 studies (8 RCTs) showed that mindfulness-based interventions (MBIs), most of which are based on Mindfulness-Based Stress Reduction (MBSR), had no apparent effect on cytokines in healthy populations. For cancer populations, MBSR was found to have some effects on cytokines, although the authors could not determine which specific cytokines were affected. Concerning IL-6, two of three studies found significant reductions following MBSR interventions (18).

Cancer-related fatigue (CaRF) is a burdensome condition that seriously impacts quality of life and is strongly associated with depression (19). Accordingly, various interventions have been employed to reduce fatigue in cancer patients (e.g., medication, exercise, rest and sleep, and psychosocial interventions) (20). As to CAM practice and its effectiveness in reducing CaRF, a systematic review of 20 studies (15 RCTs, five with quasi-experimental design) by Finnegan-John et al. (21) yielded insufficient evidence to draw conclusions.

The heterogeneous findings to date on the relationship between CAM practice and inflammatory markers and between CAM practice and CaRF may be due in part to methodological issues inherent in conventional biomedical research approaches (e.g., inadequate statistical power, diversity of cancer disease characteristics and treatment regimens, biased and heterogeneous composition of subjects, diverse biomarker examination materials, diversity with regard to CAM program frequency and duration, failure to consider the possibility of therapist effect, and usage of various forms of comparative control/placebo) (7, 18, 21). On a more basic level, we propose that the inconsistent findings are also attributable to the inadequacy of conventional research methods (e.g., pre-post design, standardized questionnaires) in grasping (a) the dynamic complexity of highly fluctuating (circulating) biomarkers such as IL-6 and (b) the complex psychosocial factors involved in the application of CAM use (e.g., personal meaning) (22–26).

The “integrative single-case design” investigating stress system dynamics in a single individual under highly realistic everyday life conditions was developed in order to capture biopsychosocial complexity (25, 27–29). Using this design, our recent publication on a breast cancer survivor provided evidence on the psychoimmunological influence of various CAM techniques experienced as positive (i.e., Jin Shin Jyutsu, music, physiotherapy, and energy healing). The patient, who suffered from depression and CaRF, engaged in CAM as part of her everyday life (23). With regard to her immune activity, statistical analysis of 55 consecutive 12-h measurements (28 days) revealed that urinary neopterin levels first increased on the day of CAM practice and then decreased after a total of 36–48 h. As cyclic responses in stress system markers are connected with meaningful everyday experiences (25) and neopterin is a reliable indicator of cellular immune activation (30, 31), this study suggests that practicing CAM was meaningful to the patient and ultimately led to inflammation reduction (23). Furthermore, CAM practice was followed by significant increases in positive mood and mental activity on the same day and then by decreases after a total of 72–84 h (positive mood) and 84–96 h (mental activity). Negative mood, by contrast, first decreased on the day of CAM practice and then increased after a total of 84–96 h following CAM.

The present article deals with the same original investigation of this breast cancer survivor and explores the impact of CAM practice on urinary IL-6 release and fatigue levels. In this regard, this study examined both the effect of each individual CAM technique practiced by the patient (i.e., Jin Shin Jyutsu, music, physiotherapy, Tai Chi, and energy healing) and the effect of all CAM techniques experienced as positive (i.e., Jin Shin Jyutsu, music, physiotherapy, and energy healing) on urinary IL-6 concentrations and fatigue. Moreover, this study investigates the dynamic interrelation between urinary IL-6 levels and fatigue.

## Case Description

The 49-year-old white woman is married and a mother of three children. She is a non-smoker and consumes alcoholic beverages moderately. The patient was diagnosed with a ductal mamma carcinoma (C50.4) of her right breast [pT2, pN1biv (6 of 13), cM0, G3, R0, ER 10%, PR 70–80%, HER2+/neu+, score = 3] 5 years before this study commenced. This stage IIB breast cancer had a 5-year survival rate at the time of diagnosis of about 85% (32). Primary cancer therapy consisted of surgery, radiotherapy, and anti-estrogen therapy (tamoxifen). Anti-estrogen therapy was finished 6 months before the study began. Given that the half-life of tamoxifen after chronic dosing is 7 days (33), the washout period for our patient ended at the latest ~4 months before study start (34). Thus, the patient's previous tamoxifen use cannot have affected the findings of this study.

With regard to her psychiatric profile, the patient has suffered chronically from dysthymia (F34.1), both before and after cancer diagnosis. Depressive symptoms increased with cancer diagnosis and therapy, leading to an additional diagnosis of adjustment disorder with depressed mood (F43.21). Moreover, with cancer diagnosis and therapy, the patient developed severe CaRF, which lasted until study start. Since cancer diagnosis, the patient has regularly applied various CAM techniques. The patient had also undergone psychotherapy for 6 months, which ended 5 months before the study began. At study start, there was no clinical evidence of metastatic or recurrent lesions. Examination before the study began revealed that the patient showed the highest score (20) on the “general fatigue” subscale of the Multidimensional Fatigue Inventory-20 (MFI-20). Moreover, the patient scored 50 out of 60 on the Center for Epidemiologic Studies Depression Scale (CES-D). In addition, on both the Spielberg STAI-S (State Trait Anxiety Inventory State) and the STAI-T (State-Trait Anxiety Inventory Trait), she had high anxiety values (78 out of 80 on STAI-S, and 69 out of 80 on STAI-T). Aspirin on an irregular basis was the only medication the patient took. Further details are given in Schubert et al. (22, 23), Haberkorn et al. (35), and Singer et al. (36).

## Materials and Methods

### Study Design

This integrative single-case study is part of a larger project on breast cancer survivors that examines the effects of emotionally meaningful incidents on stress system activity. Various results dealing with this patient have already been published (22, 23, 35, 36).

Before study start, medical check-ups and psychiatric evaluation (clinical interview, ICD-10) took place to make sure that the patient, who had suffered from breast cancer, met the inclusion and exclusion criteria specified by Bower et al. (37). The patient did not fulfill the criteria (37) in two points: She had been suffering from dysthymia (F34.1) and adjustment disorder (F43.21), both lasting until study start.

The patient was carefully instructed about sampling, storing, and transporting her urine probes. On each day of the 28-day study (from July 13th to August 9th, 2006), she collected her entire urine in a canister (containing 0.5 g Na-Metabisulfite and 0.5 g Na-EDTA to prevent urine oxidation) in two 12-h intervals per day (from ~8:00 p.m. to ~8:00 a.m. and from ~8:00 a.m. to ~8:00 p.m.), resulting in a total of 55 consecutive measurements at the end of the study. Each day, at ~8:00 a.m. and ~8:00 p.m., the patient aliquoted her 12-h urine in 2 ml Eppendorf tubes and stored the urine probes packed in labeled plastic bags in her home freezer at −20°C. Once per week, she put the 14 (2 × 7 12-h units) plastic bags in a cooling box equipped with additional ice in order to keep the temperature as low as possible so that no thawing would occur between her home and the laboratory. She was instructed to transport the frozen probes without delay from her home to the lab. There, the probes were immediately put into a biofreezer (−70°C) and stored until analysis.

Additionally, various everyday-life topics (e.g., emotional state, daily activity, routine, physical well-being) were assessed via individually tailored questionnaires (see “DIARI”) at ~8:00 a.m. and at ~8:00 p.m. each day. Once a week, the patient's general health status was examined, and the researcher conducted an interview with the patient regarding psychosocial incidents of the previous week (see “IHI”).

Further details of the study design are described in Schubert et al. (22, 23), Haberkorn et al. (35), and Singer et al. (36).

### Measurement of CAM Practice

#### Daily Inventory of Activity, Routine and Illness (DIARI)

The Daily Inventory of Activity, Routine and Illness (DIARI) (38) is used to assess a wide range of aspects related to the patient's everyday life. It consists of the following: the 3-Skalen-Version der Eigenschaftswörterliste (3-Skalen-EWL) for measurement of emotional states; several questions dealing with everyday activities and routines (e.g., physical activity, consumption of nicotine, alcohol and caffeine, sleeping habits, and medication use) and illness-related issues (e.g., possible infection, physical complaints, disturbing thoughts regarding cancer, level of fatigue, coping with cancer, and social support), most of which are determined via visual analog scales (VAS); a sheet for notes on emotionally negative and emotionally positive everyday incidents. The DIARI takes ~10 min to complete and was filled out by the patient every 12 h. For the purposes of this study, the patient's DIARI records were screened to determine the 12-h units in which she practiced CAM and how she experienced each CAM activity.

#### Incidents and Hassles Inventory (IHI)

As previously described (23), the Incidents and Hassles Inventory (IHI) (38) was conducted with the patient weekly to evaluate psychosocial everyday incidents (all types of incidents mentioned spontaneously by the patient; 39 pre-defined stressful everyday incidents; incidents experienced as negative or positive, as noted in the DIARI). All incidents as well as CAM activities were reviewed in detail (e.g., time of occurrence, extent of anticipation, meaningful people involved, memories evoked, etc). The interview was recorded on audiocassette and transcribed after the end of the study.

#### Telephone Interview and Internet Search

Due to the explorative and naturalistic character of the study, the research team was not initially aware that the patient would practice CAM during the study period. Moreover, neither the investigators nor the patient knew that CAM practice would later be evaluated for stress system and immune effects. For this reason, a telephone interview (on January 21, 2016) almost 9.5 years after the original study was conducted with the patient to retrospectively assess relevant details about the patient's CAM practice during the study period. During this interview, we learned that the patient practiced CAM not only between breast cancer diagnosis and the study start—as well as during the course of the study—but has also continued with CAM since the end of the study. In addition, the interview revealed more about the CAM techniques used by the patient during the study period. An internet search on these CAM techniques was also carried out. Further details are described in Schubert et al. (23).

#### Creation of CAM Time Series

To create the binary CAM time series from the DIARI and the weekly interviews, 12-h units in which the patient practiced CAM were coded as “1” and 12-h units with no CAM practice as “0.” Time series were created in two ways: (1) Each individual CAM technique was used to construct a time series, i.e., yielding one time series for each technique; (2) all CAM techniques experienced as positive were taken together in one time series (23).

### Measurement of Urinary Interleukin-6 per Creatinine

Urinary IL-6 concentrations were expressed in milligram per molar (mg/mol) creatinine to compensate for variations in urine density. The consecutive urinary IL-6 levels were determined through ELISA, as recommended by the manufacturer (Human IL-6 Quantikine® HS ELISA, R&D Systems, Minneapolis, MN). All 55 consecutive urinary IL-6 concentrations were measured in one single run. Urinary creatinine levels were determined using high pressure liquid chromatography (HPLC) (Model ProStar 210 Solvent Delivery Modul; Varian Associates, Palo Alto, CA), as previously described (39). Urinary creatinine levels were measured five times. A new aliquot was used for each independent run, and results were averaged.

### Measurement of Fatigue Intensity

For 12-h measurement of fatigue, a VAS was used, consisting of a 100 mm-long horizontal line and (German equivalents of) the following questions: “How would you rate your fatigue level—seen over the whole day,” “How would you rate your fatigue level—from yesterday evening until now?.” One end of the line is marked with “not at all,” the other with “strong” (German equivalents). The patient was asked to place a mark on this line to indicate the intensity of her fatigue.

### Statistical Analysis

Time series (*n* = 55) were analyzed using SPSS-TrendsTM 26.0. In order to determine if a change in one variable significantly preceded a change in another variable, and to identify temporal delays and directions of effects between the variables under investigation, cross-correlation function (CCF) was performed at lag 0 and at higher lags up to ±14. CCF was considered statistically significant when the *p* < 0.05 criterion was met.

Beforehand, time series were stationarized through differencing and/or logarithmizing whenever necessary. When auto-correlations were significant, time series were modeled using autoregressive integrated moving average (ARIMA) (40). The final models were chosen based on the information criteria [e.g., root-mean-square error (RMSE), normalized Bayesian information criterion (BIC)], the serial independency of residuals indicated by the Ljung-Box test (41) and the statistical significance of model parameters. In case of missing time series values, linear interpolation was used for correction. Additional details on the statistics applied in this study are described in Schubert et al. (25).

## Results

The time-series data set was based on 28 days, i.e., on 55 measurements (*n* = 55, no missing data). During the study period, no signs of infection were observed.

**Figure 1:**
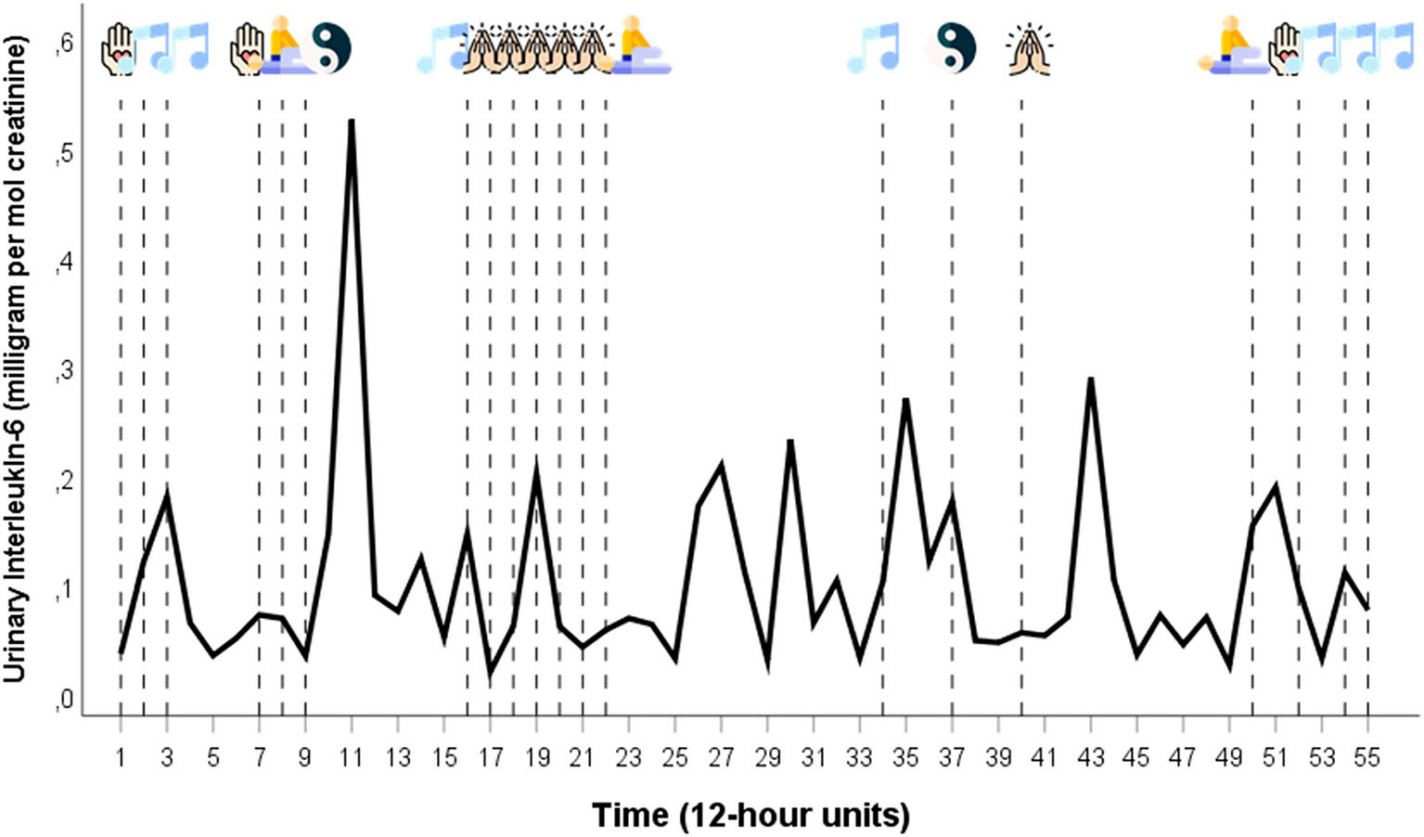
Time series of urinary IL-6 per creatinine ratios together with CAM techniques in chronological order. The time series of urinary IL-6 covers a 28-day period during which the patient collected her full urine output in 12-hour intervals. The patient practiced CAM during 21 of the 55 12-hour units. Legend of the CAM icons: 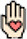 = Jin Shin Jyutsu; 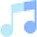 = Music; 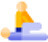 = Physiotherapy; 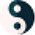 = Tai Chi; 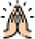 = Energy healing (Icons taken from: https://www.flaticon.com/de).

The patient practiced CAM techniques during 21 12-h units (approximately every third 12-h unit) (23). Jin Shin Jyutsu: 12-h units 1, 7, 52; music: 2, 3, 16, 34, 52, 54, 55; physiotherapy: 9, 22, 50; Tai Chi: 9, 37; energy healing: 17, 18, 19, 20, 21, 40 (Figure 1).

This study's mean values were as follows: urinary IL-6 (pg/ml): 0.6 ± 0.6 (range 0.2–2.6); urinary creatinine (μmol/l): 5.6 ± 2.5 (range 2.6–13.3); urinary IL-6/creatinine (mg/mol): 0.1 ± 0.1 (range 0.02–0.5); fatigue: 8.6 ± 0.9 (range 6.0–9.6).

Figure 1 shows the time series of urinary IL-6 per creatinine ratios together with the 21 CAM techniques in chronological order. The ARIMA models of the variables used in this study are the following: urinary IL-6/creatinine: SAR(1), s = 8; fatigue: AR(1) ln.

**Figure 2:**
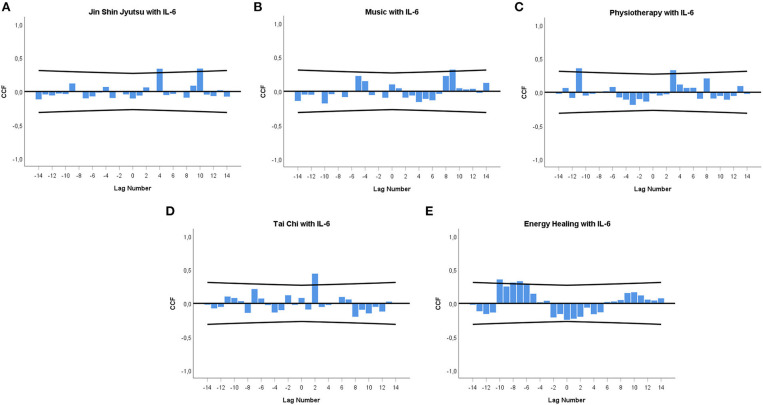
**(A–E)** Cross-correlation functions (CCFs) between individual CAM techniques and urinary IL-6 concentrations. **(A)** Jin Shin Jyutsu, **(B)** Music, **(C)** Physiotherapy, **(D)** Tai Chi, **(E)** Energy healing. Each lag represents a time interval of 12 h. Cross-correlation coefficients (bars) reaching the upper or lower limits of the 95% confidence intervals (lines) are significant at *p* < 0.05. Lag 0 on the *x*-axis signifies the time of a CAM event. A zero lag significance means that CAM is followed by an IL-6 change during the next 12 h. A positive lag significance means that CAM is followed by an IL-6 change, whereas a negative lag significance means that an IL-6 change precedes CAM.

In Figures 2A–E, the CCF between each individual CAM technique (leading variable) and the urinary IL-6 concentrations (lagging variable) are given. Throughout, CAM techniques are significantly positively cross-correlated with IL-6: Jin Shin Jyutsu: +lag 4 (*r* = +0.339; *p* = 0.012), +lag 10 (*r* = +0.341; *p* = 0.011) (Figure 2A); music: +lag 9 (*r* = +0.319; *p* = 0.018) (Figure 2B); physiotherapy: –lag 11 (*r* = +0.357; *p* = 0.008), +lag 3 (*r* = +0.329; *p* = 0.015) (Figure 2C); Tai Chi: +lag 2 (*r* = +0.442; *p* = 0.001) (Figure 2D); energy healing –lag 10 (*r* = +0.356; *p* = 0.008), –lag 8 (*r* = +0.310; *p* = 0.022), –lag 7 (*r* = +0.330; *p* = 0.014), –lag 6 (*r* = +0.287; *p* = 0.033) (Figure 2E).

With regard to the temporal connection between each CAM technique and fatigue, cross-correlational analyses revealed significantly negative associations: Jin Shin Jyutsu: +lag 4 (*r* = −0.270; *p* = 0.045); music: +lag 2 (*r* = −0.441; *p* = 0.001); physiotherapy: –lag 3 (*r* = −0.300; *p* = 0.026); Tai Chi: +lag 6 (*r* = −0.342; *p* = 0.011); energy healing –lag 2 (*r* = −0.258; *p* = 0.056, n.s.), –lag 1 (*r* = −0.255; *p* = 0.059, n.s.) (data not shown).

**Figure 3:**
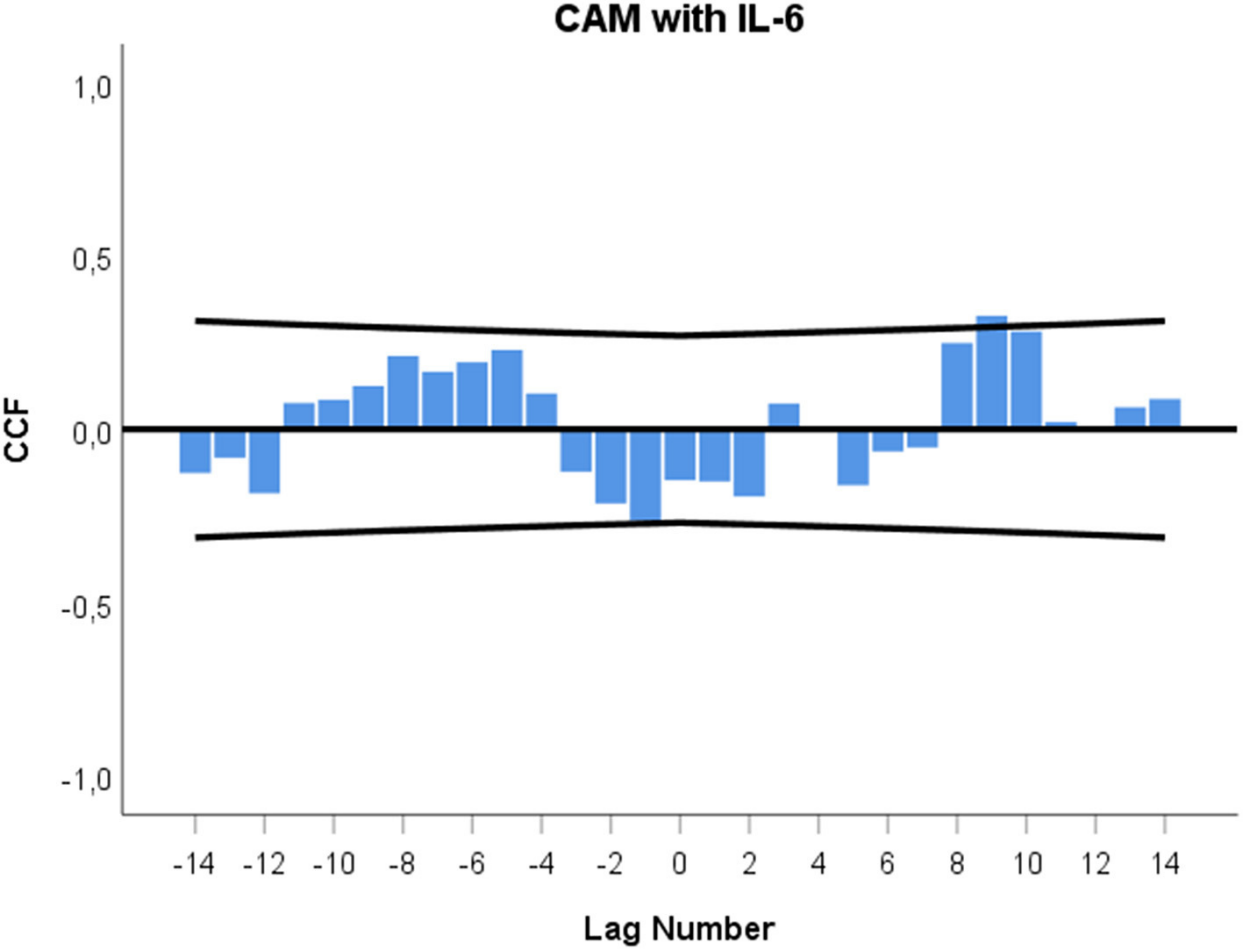
Cross-correlation function (CCF) between CAM practice experienced as positive and urinary IL-6 concentrations. See description of Figures 2A–E for explanation of graph.

Qualitative analysis revealed that Tai Chi was the only CAM technique during the study that the patient described as negative and physically stressful (23). In a next step, all CAM techniques the patient experienced as positive (i.e., Jin Shin Jyutsu, music, physiotherapy, and energy healing) were included in one time series and cross-correlated with urinary IL-6 concentrations. Figure 3 shows the result, with a negative correlation at –lag 1 (*r* = −0.265; *p* = 0.049) and a positive correlation at +lag 9 (*r* = +0.328; *p* = 0.015).

**Figure 4:**
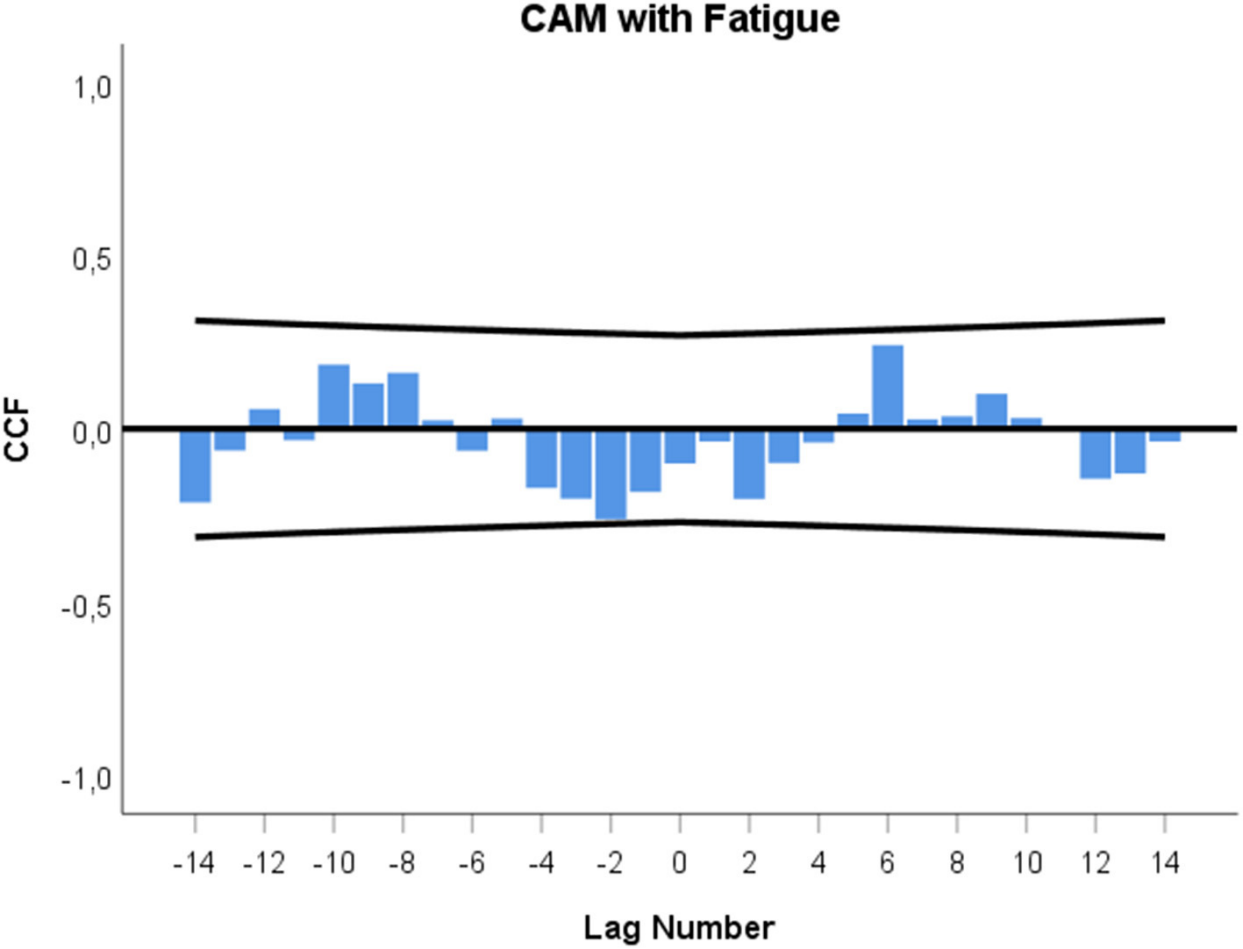
Cross-correlation function (CCF) between CAM practice experienced as positive and fatigue. Each lag represents a time interval of 12 h. Cross-correlation coefficients (bars) reaching the upper or lower limits of the 95% confidence intervals (lines) are significant at *p* < 0.05. Lag 0 on the *x*-axis signifies the time of a CAM event. A zero lag significance means that CAM is followed by a fatigue change during the next 12 h. A positive lag significance means that CAM is followed by a fatigue change, whereas a negative lag significance means that a fatigue change precedes CAM.

In Figure 4, a nearly significant cross-correlation at –lag 2 (*r* = −0.261; *p* = 0.053, n.s.) is seen between CAM techniques experienced as positive and fatigue.

**Figure 5:**
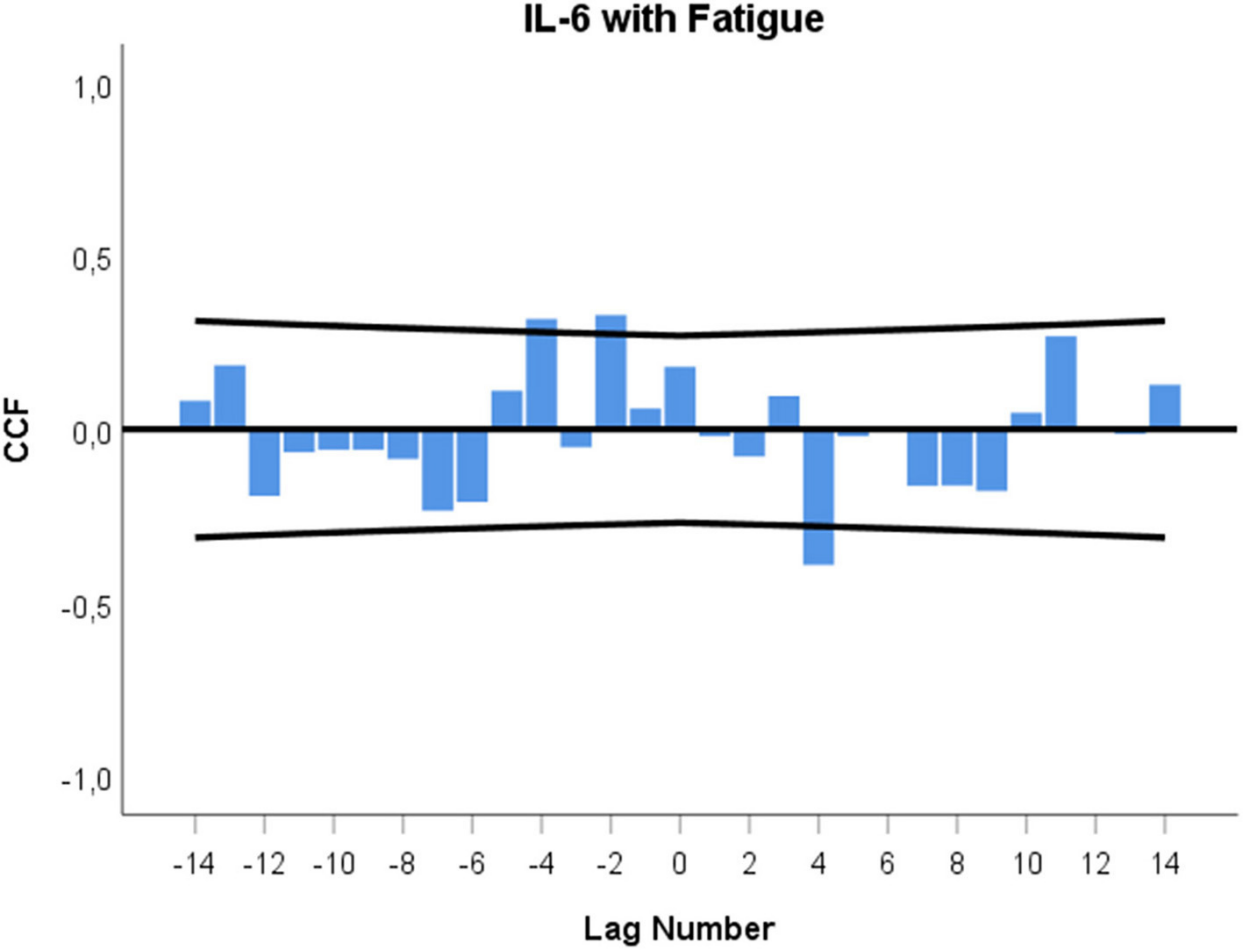
Cross-correlation function (CCF) between urinary IL-6 concentrations and fatigue levels. Each lag represents a time interval of 12 h. Cross-correlation coefficients (bars) reaching the upper or lower confidence limits (lines) are significant at *p* < 0.05. A positive lag significance means that IL-6 changes precede fatigue changes. A negative lag significance means that fatigue changes precede IL-6 changes.

Figure 5 shows the cross-correlation function between urinary IL-6 levels (leading variable) and fatigue (lagging variable). It indicates a significant negative correlation at +lag 4 (*r* = −0.392; *p* = 0.004) and significant positive correlations at –lag 2 (*r* = +0.330; *p* = 0.014) and –lag 4 (*r* = +0.318; *p* = 0.018).

## Discussion

This integrative single-case study on a breast cancer survivor suffering from CaRF and depression shows that practicing various CAM techniques (i.e., Jin Shin Jyutsu, music, physiotherapy, Tai Chi, and energy healing) affected urinary IL-6 release and fatigue intensity. The study also revealed strong interdependencies between urinary IL-6 levels and fatigue.

Specifically, it was demonstrated that each individual CAM technique was associated with significant increases of IL-6 concentrations in the patient's urine. The temporal delays between CAM practice and subsequent changes in IL-6 levels varied from 24 to 36 h (+lag 2, Tai Chi) to even 108–120 h (+lag 9, music) and 120–132 h (+lag 10, Jin Shin Jyutsu). Moreover, some increases in urinary IL-6 levels occurred in advance, i.e., up to 108–120 h (–lag 10, energy healing) or 120–132 h (–lag 11, physiotherapy) (Figures 2A–E), suggesting that the patient responded immunologically in anticipation of CAM practice. Indeed, weekly interviews revealed that several CAM techniques, such as energy healing, physiotherapy and choir rehearsals, had been planned by the patient days or even weeks in advance [see (23) for further details]. Concentration changes of stress system parameters in anticipation of the actual occurrence of an incident have repeatedly been found in integrative single-case studies (25, 28, 29). Such findings are strongly connected to the “life as it is lived” study approach, which combines thorough information on everyday incidents via qualitative methods [see (25) for further details] with statistical analysis of biopsychosocial time series data.

In addition to the findings on the temporal connection between each individual CAM technique and IL-6 changes, this study showed that when all CAM techniques experienced as positive (i.e., Jin Shin Jyutsu, music, physiotherapy, and energy healing) were taken together in one time series, a characteristic pattern emerged. Specifically, a biphasic or cyclic response pattern was identified in which urinary IL-6 levels first decreased 12–0 h preceding CAM practice and then increased a total of 108–120 h following CAM (Figure 3). This cyclic pattern of urinary IL-6 in response to CAM is similar to the pattern recently found in the same patient with regard to urinary neopterin – yet runs in the other direction. In that evaluation, urinary neopterin levels first increased on the day of CAM practice and then decreased a total of 36–48 h later (23).

Such cyclic response patterns of stress system activity belong to the central findings of integrative single-case studies. These complex patterns may be indicative of the processing of personally meaningful incidents by the stress system (25, 28) and have been detected in a variety of integrative single-case studies: in a healthy subject (25), in autoimmune patients (28, 29), in response to emotionally positive (25, 29), negative (25, 28), anticipated and unanticipated incidents (25, 28, 29), and in urinary cortisol and neopterin (25, 28). We suggest that the cyclic course of urinary IL-6 and neopterin levels [previous study (23)] observed in this patient in response to CAM practice reflects a shared unspecific operating principle [“meaning response” according to Moerman (42)] behind all the CAM activities experienced by the patient as positive (23). We further suppose that when this principle is not fully detectable, perhaps due to a low frequency of individual CAM treatments, cross-correlational analyses show only linear stress system responses, e.g., as seen in this study in the monophasic increases of urinary-IL-6 levels (Figures 2A–E).

IL-6 is a pleiotropic cytokine with pro- and anti-inflammatory properties. It orchestrates a broad spectrum of immunological and non-immunological reactions (16). We suggest that the patient's ultimate urinary IL-6 increase following CAM is an indication of anti-inflammatory action. We base our interpretation on the following observations. First, in the patient under study, CAM practice was associated with short-term positive emotional responses, i.e., increases in positive mood and mental activity as well as decreases in irritation within 12 hours following CAM practice (23). In breast cancer patients, emotionally positive occurrences have typically been connected to anti-inflammatory activity (43). Second, neopterin is released by macrophages upon stimulation of IFN-γ and indicates pro-inflammatory activity in cancer (30). In the breast cancer patient under study, neopterin ultimately *decreased* in response to CAM practice, whereas IL-6 ultimately *increased* in response to CAM. This suggests that IL-6 may be involved in functionally different—or even opposing—immunological mechanisms than neopterin (12, 15). Third, in this patient, IL-6 was followed by increases in positive emotional states and decreases in negative emotional states (22) and, as the current investigation demonstrated, decreases rather than increases in fatigue. Thus, in contrast to established thinking (8, 9), our data from this patient show that IL-6 was involved in health behavior rather than sickness behavior (22).

With regard to the temporal connection between each individual CAM technique and fatigue, a consistent decrease in fatigue was observed, with long temporal delays (up to 72–84 h, Tai Chi) and anticipatory responses (up to 24–36 h in advance, physiotherapy) (data not shown). There were no significant cross-correlations between all CAM techniques experienced as positive (i.e., Jin Shin Jyutsu, music, physiotherapy, and energy healing) and fatigue. However, when a closer look is taken at the cross-correlogram in Figure 4, a cyclic non-significant pattern between CAM practice and fatigue is seen, ranging from –lag 2 to +lag 6. This may mean that the patient's fatigue decreased 24–12 h in anticipation of CAM practice and increased 72–84 h following CAM. Such findings can be interpreted in line with the control-process view (44, 45). Namely, reduced fatigue in anticipation may be the reward in advance of CAM, while the subsequent increase in fatigue several days after CAM signaled to the patient that further CAM practice would be necessary for further health benefits.

In this study, the patient's fatigue and urinary IL-6 levels appear to be functionally interrelated in a similar manner as was found between IL-6 and emotional states in a previous investigation (22). In the current investigation, urinary IL-6 increases preceded fatigue decreases by 48–60 h, and, in the opposite direction, fatigue decreases preceded urinary IL-6 decreases by 24–36 h and 48–60 h (see Figure 5). When we take a step back from these fragmented cross-correlational entities and look at the broader picture of the dynamic interplay between IL-6 and fatigue, the cross-correlogram seen in Figure 5 shows a negative feedback loop (22, 27, 46). In this regard, Figure 5 indicates that increases in the patient's IL-6 may have initially triggered decreases in her fatigue after 48–60 h (positive lag and negative correlation in Figure 5) and that these decreased fatigue levels may have then, as per a feedback mechanism, led to decreases in IL-6 levels after a total of 24–36 h and 48–60 h (negative lag and positive correlation in Figure 5). In other words, a negative value in one course of action becomes a positive value after having interacted with the other process, and vice versa (22, 27, 46). Keeping in mind that urinary IL-6 level decreases preceded CAM practice by 12–0 h (Figure 3) and that fatigue was positively correlated with IL-6, with a temporal delay of 24–36 h (Figure 5), it may be speculated that the fatigue decrease in anticipation of CAM (Figure 4) may have had a direct influence on the IL-6 decrease in anticipation of CAM.

The theoretical basis for the negative feedback between IL-6 and fatigue shown in this study is well established. Yirmiya and Goshen (16) and Besedovsky and del Rey (47) postulate a complex circuit between central and peripheral neuro-immune activities. In their view, central immune activity triggers central neuro-endocrine responses (e.g., HPA axis, ANS) that can alter peripheral immune parameters which, in turn, influence central immune responses, creating a brain-to-body-to-brain reverberating feedback loop. In relation to IL-6, it has been shown that peripheral IL-6 can pass the blood-brain barrier via specific transporters (48) and can also activate areas of the brain that are connected to the experience of fatigue (49). The stressful experience of fatigue, moreover, may influence peripheral immune activity through neuro-endocrine transmission (50).

In future integrative single-case studies, the statistical investigation of this immune-neuro-endocrine circuit should be supplemented by multivariate time series analysis [e.g., vector autoregressive (VAR) modeling, impulse-response function (IRF) analysis] (51). This would allow the inclusion of additional variables, e.g., further stress system/immune parameters and symptoms beyond IL-6 and fatigue, in simultaneous process modeling. Another limitation of this study is the low number of occurrences of each individual CAM technique (2–7 times) and the heterogeneity of the techniques (five different techniques distributed over 28 days). These issues could be addressed in further integrative single-case studies designed with a clear focus on one specific CAM technique and its dynamic impact on stress system activity. From another perspective, however, our “life-as-it-is-lived” approach guarantees high ecological validity – a prerequisite for the generalizability of findings to real-life situations (52). In this study, the patient followed her regular everyday routine and applied CAM as a natural and meaningful part of her everyday life. For example, the patient may have known intuitively when the right time was for her to practice Jin Shin Jyutsu and music, both of which were primarily undertaken spontaneously. Indeed, all CAM practice in this study was intrinsically and not extrinsically motivated or predefined (e.g., in order to adhere to a given protocol). In this regard, in-depth interviews and qualitative analysis allowed us to identify the occurrence of CAM practice and to understand its subjective meaning for the patient.

Taken together, we suggest that the patient's intuitively chosen and intrinsically motivated CAM practice, with its corresponding immunological changes (i.e., ultimate neopterin decrease, IL-6 increase), was associated with the activation of the patient's self-healing capacities. We are convinced that only by considering essential characteristics of human life, i.e., subjective meaning and the dynamics of “life as it is lived,” can psychosomatic research evolve from biomedical to biopsychosocial (53, 54).

## Data Availability Statement

The raw data supporting the conclusions of this article will be made available by the authors, without undue reservation.

## Ethics Statement

The patient gave written informed consent to her participation and to the publication of data in accordance with the Declaration of Helsinki, and the Institutional Review Board of the Freiburg University approved the design.

## Author Contributions

MS, MO, and CS: substantial contributions to the conception or design of the work, the acquisition, analysis or interpretation of data for the work. MS, MO, HB, BH-K, FO-P, EC, and CS: drafting the work or revising it critically for important intellectual content, final approval of the version to be published, and agreement to be accountable for all aspects of the work in ensuring that questions related to the accuracy or integrity of any part of the work are appropriately investigated and resolved. All authors contributed to the article and approved the submitted version.

## Conflict of Interest

The authors declare that the research was conducted in the absence of any commercial or financial relationships that could be construed as a potential conflict of interest.
